# Histamine H2 antagonists for functional dyspepsia: A protocol for a systematic review and meta-analysis

**DOI:** 10.1097/MD.0000000000018128

**Published:** 2019-11-22

**Authors:** Juanjuan Li, Fengyun Wang, Lin Lv, Lin Xu, Enjin Zeng, Xudong Tang

**Affiliations:** a Beijing University of Chinese Medicine; b Xiyuan Hospital of China Academy of Chinese Medical Sciences; c China Academy of Chinese Medical Sciences, Beijing, China.

**Keywords:** functional dyspepsia, H_2_RA, Histamine H_2_ antagonists, protocol, systematic review

## Abstract

**Background:**

Functional dyspepsia (FD) is a prevalent gastrointestinal disorder. Histamine H_2_ antagonists (H_2_RAs) are the pharmacological treatment option for FD, but no potent evidence has been found for the efficacy of these drugs in the condition. Therefore, this systematic review protocol aims to examine the efficacy and safety of H_2_RAs in the treatment of FD.

**Methods:**

We will perform a systematic search in the following electronic databases: the Cochrane Central Register of Controlled Trials (to October 2019), MEDLINE (OvidSP; to October 2019), EMBASE (OvidSP; to October 2019). Only randomized clinical trials (RCTs) comparing any H_2_RA with placebo for the treatment of FD will be included. The primary outcome will be an improvement in global symptoms of dyspepsia. Study selection, data extraction, and study quality will be performed by 2 independent reviewers. Dichotomous data will be presented as a risk ratio (RR) with 95% confidence intervals (CI), and continuous data as mean difference (MD) or standardized MD (SMD) with 95% CI. RevMan v.5.3 software will be used for all statistical analyses.

**Results:**

This study will provide a high-quality synthesis to examine the role of H_2_RAs in FD as reflected by the improvement of global symptoms of dyspepsia, quality of life scores, and adverse events.

**Conclusion:**

This systematic review will provide updated evidence to judge whether H_2_RAs are of benefit in FD.

## Abstract 2

Supplemental Digital Content is available in the text

## 1 Introduction

Functional dyspepsia (FD) is a common but unexplained medical condition thought to originate from the gastroduodenal region. According to the Rome IV criteria, FD is divided into 2 subtypes: postprandial distress syndrome (PDS) and epigastric pain syndrome (EPS).^[1]^ PDS is characterized by meal-induced dyspeptic symptoms such as postprandial fullness and early satiety. EPS refers to bothersome epigastric pain or burning. FD affects 8% to 23% of the population in Asia,^[2]^ and accounts for 10% to 15% of the general population.^[3]^ The high prevalence of FD substantially reduces the quality of life and has significant socioeconomic consequences.^[4]^ Unfortunately, there is no definitive treatment for all individuals.^[5]^ Current management of FD focuses on symptom relief. Acid-suppressive agents such as histamine H_2_ antagonists (H_2_RAs) or proton pump inhibitors (PPIs) are commonly prescribed to patients with the condition.^[6,7]^

H_2_RAs are a group of drugs that can reduce gastric acid secretion by competitive inhibition of histamine H2 receptors located on the parietal cells.^[8]^ They have played an important role in the treatment of acid-related disorders such as gastroesophageal reflux disease and peptic ulcers.^[9]^ As for FD, The effects of H_2_RAs have been reported in several randomized clinical trials (RCTs). These trials, however, with inconsistent methodologies or outcomes may lack sufficient evidence to reach definitive conclusions.^[10]^ From 2000 to 2009, 2 meta-analyses of RCTs were published which suggested that H_2_RAs were superior to placebo in improving FD symptoms.^[6,11]^ While the results were limited due to severe methodological flaws such as the inclusion of cross-over trials, short treatment duration, and no subgroup analysis by dose of H_2_RA or H_2_RA subtype. Since then, a previous Cochrane Review has been withdrawn from publication^[12]^ and new RCTs have been developed. However, no more updated systematic reviews have been conducted. We will, therefore, perform this systematic review and meta-analysis to determine the efficacy of H_2_RAs compared with placebo in the improvement of global symptoms of dyspepsia and quality of life in FD, and to assess potential side effects as well.

## 2 Methods

### 2.1 Study registration

This systematic review protocol will adhere to the preferred reporting items for systematic reviews and meta-analysis Protocols (PRISMA-P) 2015 statement.^[13]^ Besides, The protocol is registered on the International Prospective Register of Systematic Reviews (PROSPERO registration number: CRD42019127924).

### 2.2 Criteria for considering studies for this review

#### 2.2.1 Types of studies

Any parallel-group RCTs of H_2_RA for the treatment of FD will be included. The first period of cross-over studies will be also included. Cluster-randomized trials, Quasi-RCTs will be excluded.

#### 2.2.2 Types of participants

Participants aged 18 years or over, diagnosed with FD based on either the Rome Criteria (I to IV) or a physician's opinion with a negative upper gastrointestinal endoscopy, will be included regardless of gender or race. While participants with predominant heartburn or reflux symptoms will be excluded.

#### 2.2.3 Types of interventions

Only trials comparing oral administration of any dose of H_2_RAs with placebo will be eligible for inclusion. H_2_RAs will include cimetidine, ranitidine, famotidine, nizatidine, as well as any other H_2_RAs. The minimum duration of treatment2 weeks will be included. H_2_RAs combined with any other treatment in the intervention group will be included if the combined treatment is also present in the control group.

#### 2.2.4 Types of outcome measures.

##### 2.2.4.1 Primary outcomes

The primary outcome is an improvement in global symptoms of dyspepsia, reported as a binary outcome. If global symptoms are not available, we will use epigastric pain/discomfort improvement.

##### 2.2.4.2 Secondary outcomes

- Quality of life;
- Adverse events.

### 2.3 Search methods for identification of studies

#### 2.3.1 Electronic searches

Trials will be identified by searching the following electronic databases: the Cochrane Central Register of Controlled Trials (CENTRAL) (to October 2019), MEDLINE (OvidSP) (1946 to October 2019), EMBASE (OvidSP) (1974 to October 2019). There is no language or publication status restriction. We will perform searching by using a combination of subject headings and text words. The search strategy for the MEDLINE will be shown in the Supplemental File 1, and modified by using other databases.

#### 2.3.2 Searching other resources

We will manually search conference proceedings and ClinicalTrials.gov for eligible trials. We will also check the reference lists of all studies retrieved. Besides, We will contact the authors of identified trials, manufacturers, and experts within the field to obtain further relevant studies.

### 2.4 Data collection and analysis

#### 2.4.1 Selection of studies

**Figure 1:**
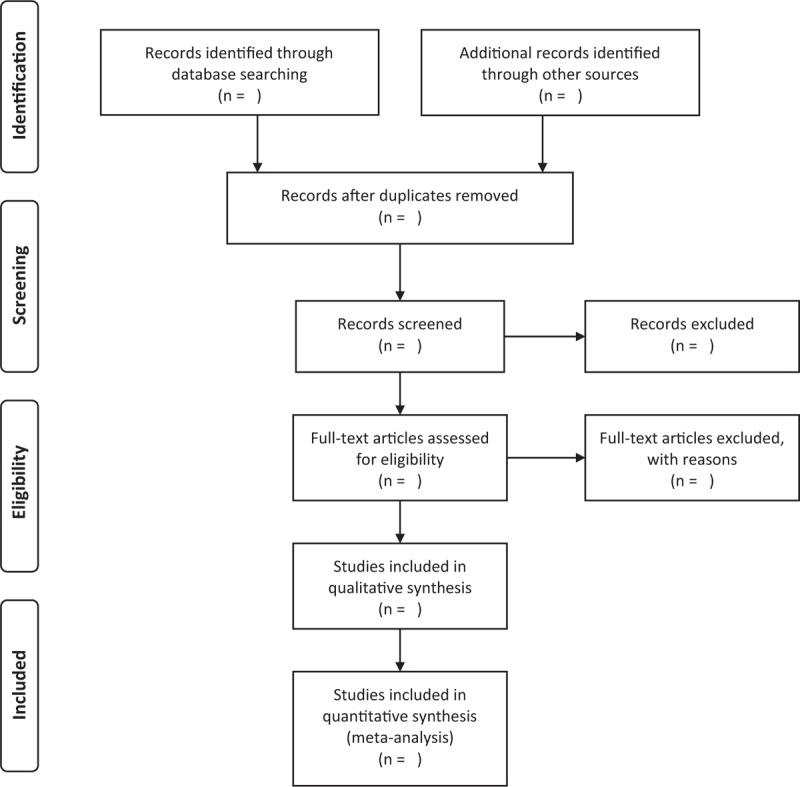
Flow diagram of the study selection process.

Studies retrieved by the search strategies will be imported and managed in the reference management software EndnoteX9. Two independent reviewers (LJJ and ZEJ) will remove duplicates and exclude irrelevant trials by screening the titles and abstracts. Then, they will review the full texts of the selected studies to determine the final included trials. Both authors will also independently collect the final data in a Microsoft Excel sheet and compare the results. Any disagreement will be resolved through discussion or by a third author (LL). The study selection process is recorded and presented in preferred reporting items for systematic reviews and meta-analysis (PRISMA) flow diagram (Fig. 1).

#### 2.4.2 Data extraction and management

We will use a specially developed form for data collection. Two review authors (XL and ZZD) will independently extract data and import it into RevMan v.5.3 software. Discrepancies will be resolved by consensus. The extracted data will include the following: the first author; publication date; study design; study setting; country of origin; sample size; diagnostic criteria used for FD; age and gender of Participants; name, dose and schedule of H_2_RA administered; duration of therapy; primary and secondary outcomes specified and collected; time points reported; withdrawals/drop-outs. Data will be extracted according to an intention-to-treat analysis.

#### 2.4.3 Assessment of risk of bias in included studies

The risk of bias in included studies will be assessed independently by 2 review authors (LJJ and ZEJ) using the Cochrane's risk of bias tool.^[14]^ There are 7 domains as follows: random sequence generation (selection bias), allocation concealment (selection bias), blinding of participants and personnel (performance bias), blinding of outcome assessment (detection bias), incomplete outcome data (attrition bias), selective reporting (reporting bias), and other bias. Each domain will be graded as high, low or unclear.

#### 2.4.4 Measures of treatment effect

The continuous outcomes will be presented as mean difference (MD) or standardized MD (SMD) with 95% confidence interval (95% CI). The binary outcomes will be presented as a risk ratio (RR) with 95% CI. Besides, we will also report the number needed to treat (NNT) and the number needed to harm (NNH), with 95% CI, according to the formula: NNT or NNH = 1/(control event rate × (1 − RR)).

#### 2.4.5 Dealing with missing data

As for the missing data, we will attempt to contact the study authors to obtain it whenever possible. If it is not available, we will perform analysis based on available data, and state how the missing data may have potential impacts on the findings in the text.

#### 2.4.6 Assessment of heterogeneity

Both the *I*^2^ statistic and the Chi^2^ test will be calculated to assess statistical heterogeneity. *I*^2^ greater than 50% or *P* value less than .1 will be considered as significant heterogeneity.^[15]^ If there is significant heterogeneity, we will perform subgroup analysis and sensitivity analysis for exploring possible sources.

#### 2.4.7 Assessment of reporting bias

A funnel plot will be constructed to identify publication bias when there are 10 or more trials. Asymmetric funnel plots suggest publication bias or small-study effects, and the results should be taken into caution. Additionally, we will also use Egger test for further quantitative analysis.^[16]^

#### 2.4.8 Data synthesis

Data synthesis will be performed by using RevMan v.5.3 from Cochrane Collaboration. We will conduct a forest plot of the meta-analysis for quantitative synthesis. If there is significant heterogeneity (*P* < .1, *I*^2^ > 50%), the random-effects model will be used for meta-analysis. Otherwise, we will consider the fixed-effects model.

#### 2.4.9 Subgroup analysis and investigation of heterogeneity

We will perform the following subgroup analysis to explore the sources of heterogeneity:

- Subtypes of FD (PDS vs EPS vs mixed type).
- Duration of therapy (<4 weeks vs ≥4 weeks).
- Dose of H_2_RA (standard-dose vs low-dose vs high-dose).
- H_2_RA subtype
- Risk of bias (low risk of bias vs unclear vs high risk of bias).

#### 2.4.10 Sensitivity analysis

Sensitivity analysis will be conducted to explore whether the results of our meta-analysis are robust. Pre-specified factors in sensitivity analysis are as follows: studies with a high risk of bias, small sample size studies, abstract inclusion, studies with the missing data.

#### 2.4.11 Grading the quality of evidence

The quality of evidence will be assessed by using the Grading of Recommendations, Assessment, Development and Evaluation (GRADE) system,^[17]^ which involves the 5 items: study limitations, consistency of effect, imprecision, indirectness, and publication bias. We will grade the quality of evidence as high, moderate, low, or very low.

## 3 Discussion

FD is a chronic and recurrent gastrointestinal disorder characterized by bothersome postprandial fullness, early satiety, epigastric pain, or burning.^[18]^ Treating FD can be challenging as a considerable overlap of symptoms and multiple mechanisms exist such as disturbed gastroduodenal motility, gastric acid secretion, and visceral hypersensitivity.^[19]^ Some evidence has suggested that a subset of FD patients respond well to acid suppression with H_2_RA or PPI therapy, even if these patients have normal gastric acid secretion.^[20]^ Unlike PPIs, H_2_RAs including cimetidine, ranitidine, famotidine, and nizatidine are not recommended as the first-line treatments for FD. Nevertheless, these drugs are widely used in clinical practice.^[21]^ Some patients even find them helpful if PPIs fail. However, the efficacy of H_2_RAs in FD remains controversial.

We will perform this systematic review of H_2_RAs for the treatment of FD to inform patients, clinicians, and policymakers of the efficacy and safety of this medication. However, there may be potential limitations to this research. First, inter-study variability in the diagnosis of FD, country of origin, sample size, and definition of symptom improvement may contribute to heterogeneity risks. Second, the quality of trials likely affects the reliability of the final results.

## Author contributions

**Conceptualization:** Fengyun Wang, Xudong Tang

**Data curation:** Juanjuan Li, Lin Xu, Enjin Zeng

**Formal analysis:** Lin Lv

**Investigation:** Lin Xu, Enjin Zeng

**Supervision:** Lin Lv

**Writing – original draft:** Juanjuan Li

**Writing – review and editing:** Juanjuan Li

juanjuan li orcid: 0000-0003-4581-3788.

## Supplementary Material

Supplemental Digital Content
