# Supplementary material for: Histamine H2 antagonists for functional dyspepsia: A protocol for a systematic review and meta-analysis
Source: Medicine (Baltimore). 2019 Nov 22;98(47):e18128. doi: 10.1097/MD.0000000000018128 (PMC6882590; doi:10.1097/MD.0000000000018128)
Supplement: Supplemental Digital Content [file medi-98-e18128-s001.doc]

Supplemental File 1. MEDLINE (OvidSP) search strategy

| Number | Search terms |
| --- | --- |
| #1 | exp Dyspepsia/ |
| #2 | (Dyspepsia or dyspeptic or NUD or FD).mp. |
| #3 | (indigestion or indigestive).tw. |
| #4 | or/1-3 |
| #5 | exp Histamine H_2_ receptor antagonist/ |
| #6 | ((H_2_ adj2 receptor adj2 antagonist$) or (histamine adj2 H_2_ adj2 antagonist$) or H_2_RA or H_2_RAs).tw. |
| #7 | exp ranitidine/ |
| #8 | (Ranitidine or Biotidin or Histodil or Tagamet or Altramet or Eureceptor).tw. |
| #9 | exp cimetidine/ |
| #10 | (Cimetidine or Biomet or Ranisen or Sostril or Zantac or Zantic).tw. |
| #11 | exp famotidine/ |
| #12 | (Pepcid or Famotidine).tw. |
| #13 | exp nizatidine/ |
| #14 | (Axid or Nizatidine).tw. |
| #15 | or/5-14 |
| #16 | #4 and #15 |
| #17 | randomized controlled trial.pt. |
| #18 | controlled clinical trial.pt. |
| #19 | randomized.ab. |
| #20 | placebo.ab. |
| #21 | drug therapy.fs. |
| #22 | randomly.ab. |
| #23 | trial.ab. |
| #24 | groups.ab. |
| #25 | or/17-24 |
| #26 | #16 and #25 |
| #27 | exp animals/ not humans.sh. |
| #28 | 26 not 27 |
